# Immune response and severity of Omicron BA.5 reinfection among individuals previously infected with different SARS-CoV-2 variants

**DOI:** 10.3389/fcimb.2023.1277880

**Published:** 2023-12-22

**Authors:** Lu Li, Zhiwei Xie, Youxia Li, Minhan Luo, Lieguang Zhang, Chengqian Feng, Guofang Tang, Huang Huang, Ruitian Hou, Yujuan Xu, Shijie Jia, Jingrong Shi, Qinghong Fan, Qingxin Gan, Na Yu, Fengyu Hu, Yueping Li, Yun Lan, Xiaoping Tang, Feng Li, Xilong Deng

**Affiliations:** ^1^ Institute of Infectious Diseases, Guangzhou Eighth People’s Hospital, Guangzhou Medical University, Guangzhou, China; ^2^ Department of Critical Care Medicine, Guangzhou Eighth People’s Hospital, Guangzhou Medical University, Guangzhou, China; ^3^ Department of Radiology, Guangzhou Eighth People’s Hospital, Guangzhou Medical University, Guangzhou, China; ^4^ Department of Traditional Chinese Medicine, Guangzhou Eighth People’s Hospital, Guangzhou Medical University, Guangzhou, China; ^5^ Guangzhou Laboratory, Bio-Island, Guangzhou, China; ^6^ Department of Infectious Critical Care Medicine, Guangzhou Eighth People’s Hospital, Guangzhou Medical University, Guangzhou, China

**Keywords:** reinfection, Omicron BA.5, immune response, SARS-COV-2 variants, disease severity

## Abstract

**Introduction:**

COVID-19 continues to spread worldwide, with an increasing number of individuals experiencing reinfection after recovering from their primary infection. However, the nature and progression of this infection remain poorly understood. We aimed to investigate the immune response, severity and outcomes of Omicron BA.5 reinfection among individuals previously infected with different SARS-CoV-2 variants.

**Methods:**

We enrolled 432 COVID-19 cases who had experienced prior infection with the ancestral SARS-CoV-2 virus, Delta variant or Omicron BA.2 variant between January 2020 and May 2022 in Guangzhou, China. All cases underwent follow-up from March to April, 2023 through telephone questionnaires and clinical visits. Nasal lavage fluid and peripheral blood were collected to assess anti-RBD IgA, anti-RBD IgG and virus-specific IFN-γ secreting T cells.

**Results:**

Our study shows that 73.1%, 56.7% and 12.5% of individuals with a prior infection of the ancestral virus, Delta or Omicron BA.2 variant experienced reinfection with the BA.5 variant, respectively. Fever, cough and sore throat were the most common symptoms of BA.5 reinfection, with most improving within one week and none progressing to a critical condition. Compared with individuals without reinfection, reinfected patients with a prior Delta infection exhibited elevated levels of nasal anti-RBD IgA, serum anti-RBD IgG and IFN-γ secreting T cells, whereas there was no noticeable change in reinfected individuals with a prior BA.2 infection.

**Conclusion:**

These results suggest that BA.5 reinfection is common but severe outcomes are relatively rare. Reinfection with a novel SARS-CoV-2 variant different from the prior infection may induce a more robust immune protection, which should be taken into account during vaccine development.

## 1 Introduction

Since the start of the coronavirus disease 2019 (COVID-19) pandemic, the possibility of reinfection has remained a constant concern (Islam, 2023). This concern has grown especially pronounced with the emerge of the SARS-CoV-2 Omicron variant, which shows increased transmissibility, altered disease severity, and potential immunity evasion, consequently leading to a rise in reported cases of reinfection with different variants (Pulliam et al., 2022; Tan et al., 2023). According to the WHO, antibodies in recovered patients does not guarantee protection from reinfection, especially in patients older than 65 years old (World Health Organization, 2021). As the virus continues to mutate, patients who have previously been infected may face reinfection with new variants. It is import to know how the clinical manifestations and immune responses of individuals who have previously been infected with different virus strains differ when exposed to the same epidemic strain.

Because of the early implementation of Dynamic Zero-COVID Policy in China, incidences of SARS-CoV-2 reinfections were rarely reported. However, between December 2022 and January 2023, the prevalence of COVID-19 surged, coinciding with dominance of the BA.5.2 variant and its sub-lineages in Guangzhou, China (China CDC weekly, 2023). It is unclear that whether previously infected cases are reinfected in this epidemic and what are the effects of primary infection with different virus strains on reinfection. Understanding this information will help to provide preventive assistance to people with different infection backgrounds.

In this study, we aimed to characterize the severity, outcomes and immunological characteristics of SARS-CoV-2 reinfection in individuals who had a primary infection with different SARS-CoV-2 variants. We investigated a total of 432 cases who were classified into ancestral SARS-CoV-2, Delta and Omicron BA.2 groups according to their original infecting strains. We anticipate that our findings will shed light on the complex interplay between the host immune system and the evolving virus, thereby enhancing our understanding of the nature and progression of the disease. This research carries significant importance in comprehending the impact on public health, providing guidance to healthcare workers in clinical management of COVID-19, and informing future therapeutic interventions and vaccination strategies.

## 2 Materials and methods

### 2.1 Study design and participants

This research was a retrospective study of 432 patients with confirmed COVID-19 who were admitted to Guangzhou Eighth People’s Hospital from January 2020 to May 2022. They were categorized into the ancestral SARS-CoV-2, Delta and Omicron BA.2 groups based on the strains with which they were initially infected. All cases underwent follow-up from March to April, 2023 through telephone questionnaires and clinical visits. BA.5 reinfection occurred between December 2022 and January 2023 in some of these cases according to the telephone questionnaires. As a control for reinfection with BA.5, 33 cases with BA.5 primary infection were included.

To compare the immune response and severity of Omicron BA.5 reinfection among individuals previously infected with different SARS-CoV-2 variants, clinical information and specimen were collected after experiencing reinfection. Peripheral blood and nasal lavage fluid (NALF) samples were collected to detect the levels of anti-RBD-IgA, anti-RBD-IgG antibodies and specific IFN-γ^+^ T cell response. Patients with incomplete telephone questionnaires or clinical visits were excluded.

The study was reviewed and approved by the ethical committee of Guangzhou Eighth People’s Hospital, Guangzhou Medical University, China (No. 202115202 and No. 202305242). A written consent was obtained from either each patient or their next of kin in this study.

### 2.2 Identification of SARS-CoV-2 strains in initial infection

All cases included in this study were local COVID-19 patients in Guangzhou as previously reported (Wang et al., 2021; Li et al., 2022; Li et al., 2023). The virus strains they were initially infected with were identified by whole viral genome sequencing. During hospitalization for their initial infection, specimens were collected, and subjected to whole viral genome sequencing by the Nanopore technology (Oxford Nanopore, UK). Sequencing libraries were prepared using the amplicon-based enrichment method as described previously (Yan et al., 2021) and the Ligation Sequencing Kit (Oxford Nanopore, UK, Cat No: SQK-LSK109). Sequencing was performed on the Nanopore MK1B platform. Raw sequencing data was collected using the ONT MinKNOW software and analyzed by Guppy in local base calling. Only reads with a length of at least 350 bp were selected for viral whole genome assembly. Virus lineage assignment was determined by submitted the whole genome assemblies to the Pangolin COVID-19 Lineage Assigner (https://pangolin.cog-uk.io/).

### 2.3 Definition of reinfection

According to the 10^th^ Diagnosis and Treatment Protocol for COVID-19 issued by Chinese National Health Commission and the COVID-19 Case Definition published by the U.S. Centers for Disease Control and Prevention (CDC) (U.S. Centers for Disease Control and Prevention, 2021; National Health Commission of the People’s Republic of China, 2023), reinfection cases were categorized as confirmed, probable and suspect cases. Confirmed cases were defined as those 1) who had a positive test result for SARS-CoV-2 nucleic acid irrespective of symptoms or 2) who had a positive test for SARS-CoV-2 specific antigen with related symptoms. Probable cases were those 1) who met clinical criteria and epidemiologic linkage with no SARS-CoV-2 nucleic acid or antigen test or 2) who tested positive for SARS-CoV-2 specific antigen. Suspect cases were defined as those whose NALF is positive for RBD-IgA during follow-up. In this study, reinfection cases included all confirmed, probable and suspect cases.

The clinical criteria included meeting at least one condition of the following: 1) acute onset or worsening of any one of the following symptoms or signs: cough, shortness of breath, difficulty breathing, olfactory disorder, and taste disorder; 2) acute onset or worsening of at least two of the following symptoms or signs: fever, sore throat, congestion or runny nose, muscle or joint pain, headache, fatigue, nausea or vomiting, and palpitation. The epidemiologic linkage meant close contact with a confirmed or probable case of COVID-19 disease in the prior 14 days.

The virus type responsible for reinfections in these patients was not determined by viral genome sequencing but predicted to primarily involve the BA.5.2 variant as this variant accounted for >85% of reported COVID-19 cases in Guangdong Province between December 1st, 2022 and January 9th, 2023 according to surveillance data from the Chinese Center for Disease Control and Prevention (Chinese Centre for Disease Control and Prevention, 2023).

### 2.4 Clinical data collection

Demographic data, clinical symptoms and signs, and laboratory findings of all patients during their initial infection were collected from the hospital information system. These patients were followed-up for reinfection through telephone questionnaires and clinical visits. During the clinical visits, clinical information were collected by medical professionals through interviews and questionnaires. Patients with incomplete telephone questionnaires or clinical visits were excluded. Vaccination record was obtained from the Public Health Department of the WeChat mini-program (Tencent, China).

### 2.5 Detection of anti-RBD antibodies

Serum anti-RBD IgG antibody was detected using the two-step Indirect Immunoassay Electrochemiluminescence Immunoassay Kit (Antu Biotech Co., Ltd.) as previously reported (Fan et al., 2022). Cut off index (COI) is the unit of results which represents the ratio of the detected optical intensity value to the threshold value. If COI is less than 1, the result is negative. Anti-RBD IgA antibody in NALF was detected using the chemiluminescence immunoassay kit (Beijing Savant Biotechnology Co., ltd.).

### 2.6 IFN-γ ELISpot assay

The IFN-γ ELISpot assay was used to determine the T cell response to SARS-CoV-2 as we previously described (Feng et al., 2021). Briefly, 96-well filter plates were coated with anti-IFN-γ mAb (U-Cytech, Netherlands, No. CT640-10) overnight at 4°C. Then wells were washed and blocked for 2 hours at 37°C. Freshly isolated PBMCs from patients were plated and stimulated by peptide pools of nucleocapsid (N), Spike 1 sub-unit (S1) or 2 (S2) of SARS-CoV-2 ancestral strain for 24h at 37°C. Following washing with PBS-Tween 20, the plates were incubated with biotinylated anti-IFN-γ detection antibody and alkaline phosphatase-conjugated streptavidin. Subsequently, the plates were incubated with NBT/BCIP (Pierce, USA) for 10 minutes. Spot numbers of IFN-γ-secreting cells were counted using the ELISPOT reader (Bioreader 4000, BIOSYS, Germany).

### 2.7 Statistical analysis

Baseline characteristics were summarized as categorical variables, and expressed as frequencies and percentages (%). The Fisher exact test or χ^2^ test was used to compare categorical variables. Continuous variables were presented as mean ± standard deviation (SD) or median and inter-quartile range (IQR). Independent t-test or Mann-Whitney U test was used to compare continuous variables, as appropriate. The data were analyzed using SPSS software (version 25.0; IBM). A *P*-value < 0.05 was considered statistically significant.

## 3 Results

### 3.1 Demographic and clinical characteristics of subjects during the primary infection

**Figure 1:**
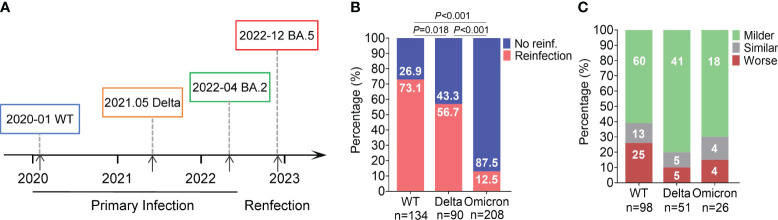
BA.5 reinfection rate and disease severity in individuals with primary infection with different variants. **(A)** Epidemic timeline of different primary infection and reinfection variants of SARS–CoV–2. **(B)** Incidence of BA.5 reinfection. Percentages were showed in the column. **(C)** Changes in clinical severity of BA.5 reinfection compared to primary infection. Case number were showed in the column.

During the COVID-19 pandemic period, patients infected with various virus strains in Guangzhou were admitted to the Guangzhou Eighth People’s Hospital, Guangzhou Medical University, China. The ancestral strain was first reported on January 20, 2020, and the Delta and Omicron BA.2 variants primarily emerged on May 21, 2021 and April 8, 2022, respectively (Wang et al., 2020; Wang et al., 2021; Li et al., 2023). Some of these cases reinfected with the Omicron BA.5 variant between December, 2022 and January, 2023 according to the telephone questionnaires (**Figure 1A**).

In this study, a total of 432 individuals from Guangzhou were included and classified into three groups based on the SARS-CoV-2 strain in their prior infection: 134 in the ancestral SARS-CoV-2 group (referred to as the WT group), 90 in the Delta group and 208 in the Omicron BA.2 group (BA.2 group). The additional 33 cases with BA.5 primary infection were included as controls.

**Table 1** illustrates the demographic and clinical characteristics of these participants during their primary infection. The BA.2 group were significantly younger compared to the WT and Delta groups (*P* < 0.001). Approximately 60% of participants in the WT and Delta groups were aged 18-60 years old, with over 30% being above 60 years old. Conversely, more than 86% were aged 18-60 years old in the BA.2 group. The gender distribution did not display any significant variation across the three groups (*P* > 0.05). However, a statistically significant difference in disease severity was observed among the three groups (*P* < 0.001). The WT and Delta groups primarily consisted of moderate cases, whereas over 90% were asymptomatic or mild cases in the BA.2 group. In terms of vaccination history, 71.6% of participants in the BA.2 group received three doses of vaccine, significantly compared to the WT and Delta groups (*P* < 0.001). Furthermore, 32.8% (44/134) of the WT group, 26.7% (24/90) of the Delta group and 24.0% (50/208) of the BA.2 group had comorbidities, but with no significant differences (*P*=0.202).

**Table 1:** Demographic and clinical characteristics of subjects during the initial infection.

	Variant type in the initial infection (n = 460)								P
	WT (n = 134)		Delta variant (n = 90)		BA.2variant (n = 208)		BA.5 infection (n=33)		
Age									
Median(P_25_–P_75_), yr	51	(38–62)	49	(35–65)	40	(27–50)^*,#^	45.0	(33–53)	<0.001
<18, n (%)	0	(0.0)	10	(11.1)^*^	21	(10.1)^*^	0	(0.0)	<0.001
18≤age ≤ 60, n (%)	91	(67.9)	51	(56.7)	180	(86.5)^*,#^	33	(100)^*,#^	
>60, n (%)	43	(32.1)	29	(32.2)	7	(3.4)^*,#^	0	(0.0)^*,#^	
Gender, n (%)									
Male	62	(46.3)	37	(41.1)	102	(49.0)	10	(30.3)	0.183
Female	72	(53.7)	53	(58.9)	106	(51.0)	23	(69.7)	
Severity of initial infection, n(%)									
Asymptomatic or Mild	9	(6.7)	18	(20.0)^*^	188	(90.4)^*,#^	–	–	<0.001
Moderate	100	(74.6)	63	(70.0)	20	(9.6)^*,#^	–	–	
Severe or Critical	25	(18.7)	9	(10.0)	0	(0.0)^*,#^	–	–	
Vaccination status^&^, n (%)									
Vaccinated	103	(76.9)	68	(75.6)	188	(90.4)^*,#^	33	(100)^*,#^	<0.001
0 Dose	30	(22.4)	22	(24.4)	4	(1.9)^*,#^	0	(0)^*,#^	<0.001
1 Dose	5	(3.7)	16	(17.8)^*^	7	(3.4)^#^	0	(0)	
2 Doses	50	(37.3)	35	(38.9)	30	(14.4)^*,#^	7	(21.2)	
3 Doses	48	(35.8)	16	(17.8)^*^	149	(71.6)^*,#^	24	(72.7)^*,#^	
4 Doses	0	(0.0)	1	(1.1)	2	(1.0)	2	(6.1)^*^	
No record	1	(0.7)	0	(0.0)	16	(7.7)	0	(0)	–
Comorbidity, n (%)									
Occurrence	44	(32.8)	24	(26.7)	50	(24.0)	–	–	0.202
Hypertension	24	(17.9)	11	(12.2)	17	(8.2)^*^	–	–	0.026
Cardiac Disease	9	(6.7)	2	(2.2)	3	(1.4)^*^	–	–	0.027
Diabetes	4	(3.0)	6	(6.7)	5	(2.4)	–	–	0.185
Lung Disease	8	(6.0)	4	(4.4)	8	(3.9)	–	–	0.659
Liver Disease	7	(5.2)	2	(2.2)	17	(8.2)	–	–	0.126
Renal Disease	3	(2.2)	2	(2.2)	6	(2.9)	–	–	>0.999
Mental Disease	0	(0.0)	0	(0.0)	1	(0.5)	–	–	>0.999
Thyroid Disease	3	(2.2)	4	(4.4)	3	(1.4)	–	–	0.281
Others	0	(0.0)	0	(0.0)	5	(2.4)	–	–	0.109
No record	6	(4.5)	2	(2.2)	19	(9.1)	33	(100)	–
Reinfection, n (%)									
Non–Reinfection	36	(26.9)	39	(43.3)	182	(87.5)^*,#^	33	(100)^*,#^	<0.001
Reinfection	98	(73.1)	51	(56.7)	26	(12.5)^*,#^	0	(0.0)^*,#^	
Confirmed	9	(9.18)	2	(3.92)	3	(11.54)	–	–	0.215
Probable	83	(84.69)	41	(80.39)	22	(84.62)	–	–	
Suspect	6	(6.12)	8	(15.69)	1	(3.85)	–	–	

Data are presented as median (P_25_–P_75_) or number (%). P values were determined using the Mann–Whitney U–test for continuous variables and ordered data, and the Chi–square test for categorical variables. In pairwise comparisons among the three groups of data, Holm–Bonferroni correction was applied for continuous variables and ordered data, while Bonferroni correction was applied for categorical variables.

P represents the statistical significance among the 3 groups. * P-values compared with WT group: *P < 0.05, **P < 0.01, ***P < 0.001. # P-values compared with Delta variant group: # P < 0.05, ## P < 0.01, ### P < 0.001.

^&^Here shows the vaccination status before reinfection according to cases’ vaccination record from the Public Health Department of the WeChat mini–program or qustionnaire. All vaccines taken are inactivated SARS–CoV–2 virus vaccines.

### 3.2 Comparison of demographic and clinical characteristics between patients with reinfection and those without

Among 432 patients, 175 (40.5%) and 257 (59.5%) were identified and divided into SARS-CoV-2 reinfection and non-reinfection cohorts, respectively. A comparison of the demographic and clinical characteristics between the two cohorts is displayed in **Table 2**. No factors were found to have a significant impact on reinfection rates across the three groups, except for the gender distribution (*P*=0.046) and vaccination status (*P*=0.048) in the BA.2 group. Interestingly, individuals with lung disease within the WT group exhibited a reduced likelihood of COVID-19 reinfection (*P*=0.039); however, this phenomenon was not observed within the Delta and Omicron groups. The reinfection interval since primary infection significantly differed among the three groups, with 100% were >24 months in WT reinfection group, 94.12% were 18-24 months in Delta reinfection group and 96.15% were 6-12 months in BA.2 reinfection group (*P*<0.01, data not shown), showing three distinct waves of SARS-CoV-2 infections attributable to the respective strains. Moreover, there were no significant differences in occurrence of comorbidities between reinfections and non-reinfections among three groups, indicating that comorbidity is not a key factor affecting reinfection.

**Table 2:** Clinical characteristics of patients reinfected with SARS–CoV–2.

	WT infection (n=134)				*P*	Delta infection (n=90)				*P*	BA.2 infection (n=208)				*P*
	Reinfection (n=98)		Non–Reinf. (n=36)			Reinfection (n=51)		Non–Reinf. (n=39)			Reinfection (n=26)		Non–Reinf.(n=182)		
Age															
Median(P_25_–P_75_), yr	50	(40–61)	55	(36–63)	0.668	54	(40–65)	48	(33–67)	0.605	36	(29–47)	40	(29–46)	0.965
<18, n(%)	0	(0.0)	0	(0.0)	0.064	5	(9.8)	5	(12.8)	0.258	0	(0.0)	21	(11.5)	0.284
18≤age ≤ 60, n(%)	71	(72.5)	20	(55.6)		27	(52.9)	24	(61.6)		26	(100.0)	154	(84.6)	
>60, n(%)	27	(27.5)	16	(44.4)		19	(37.3)	10	(25.6)		0	(0.0)	7	(3.9)	
Gender, n(%)															
Male	41	(41.8)	21	(58.3)	0.090	18	(35.3)	19	(48.7)	0.280	8	(30.8)	94	(51.7)	0.046
Female	57	(58.2)	15	(41.7)		33	(64.7)	20	(51.3)		18	(69.2)	88	(48.3)	
Classification of Primary Infection, n(%)															
Asymptomatic or Mild	6	(6.1)	3	(8.3)	0.359	9	(17.6)	9	(23.1)	0.413	25	(96.2)	163	(89.6)	0.287
Moderate	72	(73.5)	28	(77.8)		36	(70.6)	27	(69.2)		1	(3.8)	19	(10.4)	
Severe or Critical	20	(20.4)	5	(13.9)		6	(11.8)	3	(7.7)		0	(0.0)	0	(0.0)	
Reinfection Interval^*^ Since Primary Infection															
6–12 Months	0	(0.0)	0	(0.0)	>0.999	0	(0.0)	0	(0.0)	0.255	25	(96.2)	182	(100.0)	0.100
12–18 Months	0	(0.0)	0	(0.0)		3	(5.9)	0	(0.0)		1	(3.8)	0	(0.0)	
18–24 Months	0	(0.0)	0	(0.0)		48	(94.1)	39	(100.0)		0	(0.0)	0	(0.0)	
>24 Months	98	(100.0)	36	(100.0)		0	(0.0)	0	(0.0)		0	(0.0)	0	(0.0)	
Reinfection Interval^**^ Since Last Vaccination, Median(P_25_–P_75_), day															
Time Interval	342	(288–406)	383	(296–540)	0.320	265	(230–288)	260	(173–565))	0.907	365	(312–435)	363	(305–430)	0.809
Vaccination^***^ Status, n(%)															
Vaccinated	79	(80.6)	24	(66.7)	0.143	38	(74.5)	30	(76.9)	0.811	23	(88.5)	165	(90.7)	>0.999
0 Dose	19	(19.4)	11	(30.6)	0.317	13	(25.5)	9	(23.1)	0.714	0	(0.0)	4	(2.2)	0.048
1 Dose	5	(5.1)	0	(0.0)		9	(17.6)	7	(18.0)		0	(0.0)	7	(3.9)	
2 Doses	36	(36.7)	14	(38.9)		20	(39.3)	15	(38.4)		1	(3.9)	29	(15.9)	
3 Doses	38	(38.8)	10	(27.8)		9	(17.6)	7	(18.0)		22	(84.6)	127	(69.8)	
4 Doses	0	(0.0)	0	(0.0)		0	(0.0)	1	(2.5)		0	(0.0)	2	(1.1)	
No Info.	0	(0.0)	1	(2.8)	–	0	(0.0)	0	(0.0)	–	3	(11.5)	13	(7.1)	–
Comorbidity, n(%)															
Occurrence	28	(28.6)	16	(44.4)	0.151	16	(31.4)	8	(20.5)	0.253	6	(23.1)	44	(24.2)	>0.999
Hypertension	17	(17.4)	7	(19.4)	>0.999	7	(13.7)	4	(10.3)	0.751	1	(3.9)	16	(8.8)	0.699
Cardiac Disease	4	(4.1)	5	(13.9)	0.116	1	(2.0)	1	(2.6)	>0.999	0	(0.0)	3	(1.7)	>0.999
Diabetes	4	(4.1)	0	(0.0)	0.576	3	(5.9)	3	(7.7)	>0.999	0	(0.0)	5	(2.8)	>0.999
Lung Disease	3	(3.1)	5	(13.9)	0.039	3	(5.9)	1	(2.6)	0.631	1	(3.9)	7	(3.9)	>0.999
Liver Disease	5	(5.1)	2	(5.6)	>0.999	2	(3.9)	0	(0.0)	0.504	1	(3.9)	16	(8.8)	0.699
Renal Disease	2	(2.0)	1	(2.8)	>0.999	1	(2.0)	1	(2.6)	>0.999	0	(0.0)	6	(3.3)	>0.999
Mental Disease	0	(0.0)	0	(0.0)	>0.999	0	(0.0)	0	(0.0)	>0.999	1	(3.9)	0	(0.0)	0.122
Thyroid Disease	3	(3.1)	0	(0.0)	0.559	3	(5.9)	1	(2.6)	0.631	0	(0.0)	3	(1.7)	>0.999
Others	0	(0.0)	0	(0.0)	>0.999	0	(0.0)	0	(0.0)	>0.999	2	(7.7)	3	(1.7)	0.113
No Info.	6	(6.1)	0	(0.0)	–	1	(2.0)	1	(2.6)	–	3	(11.5)	16	(8.8)	–
Interval between reinfection and clinical specimen collection															
Median(P_25_–P_75_), day	109	(106–114)	–	–		105	(95–117.5)	–	–		104	(99.3–144.3)	–	–	0.180
Therapy during reinfection, n(%)															
No drugs	37	(37.8)				25	(49.0)				16	(57.1)			0.071
Cold medicine	38	(38.8)				8	(15.7)				4	(14.3)			0.012
Traditional Chinese patent medicines	14	(14.3)				14	(27.5)				6	(21.4)			
Both cold and traditional Chinese medicine	9	(9.2)				4	(7.8)				0	(0.0)			

Data are presented as median (P_25_–P_75_) or number (%). P values were determined using the Mann–Whitney U–test for continuous variables and ordered data, and the Chi–square test for categorical variables.

“−” means not applicable.

* and **For nonreinfection group: Instead of reinfection date, the median dates of reinfection groups (2022–12–15 for Delta and WT reinfection, and 2022–12–20 for BA.2 reinfection) are employed as the end date of time interval.

***All vaccines taken are inactivated vaccines for SARS–CoV–2.

### 3.3 Clinical symptoms of Omicron BA.5 reinfection

**Figure 2:**
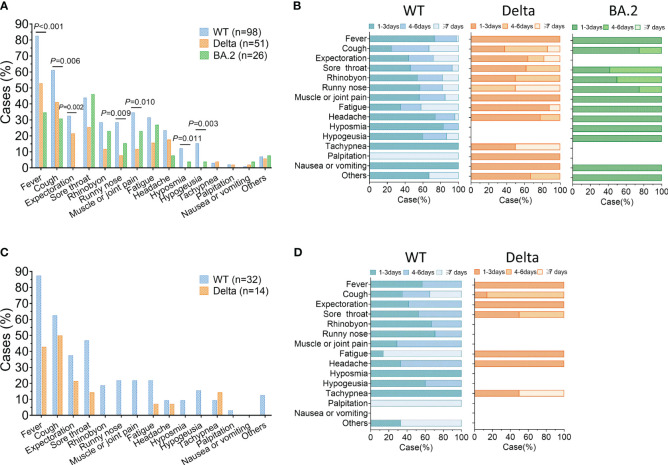
Clinical symptoms of Omicron BA.5 reinfection. **(A)** Prevalence of symptoms reported in reinfection. **(B)** Duration of clinical symptoms in reinfection. **(C, D)** Prevalence and duration of symptoms during reinfection of the severe/critical cases in primary infection or cases older than 65. WT = wild type.

Firstly, we compared the incidence of reinfection between the three group. The reinfection rate was 73.13% (98/134) in the WT group, 56.67% (51/90) in the Delta group, and 12.50% (26/208) for the BA.2 group, with significantly differences among the three groups (**Figure 1B**). Compared to primary infection, 61% (60/98) of ancestral virus, 80% (41/51) of Delta, and 70% (18/26) of BA.2 group experience milder symptoms during BA.5 reinfection (**Figure 1C**). Furthermore, we would like to know how the outcomes of BA.5 reinfection differed among the three groups. We analyzed the specific reinfection symptoms. The common symptoms included fever, cough, expectoration, sore throat, rhinobyon, runny nose, and muscle or joint pain. The incidence of these common symptoms varied among the three groups. For prior ancestral virus or Delta infection, the top three frequently symptoms of BA.5 reinfection were fever (82.65% or 52.94%), cough (61.22% or 41.18%) and sore throat (43.88% or 25.49%), different from previous BA.2 infection group whose most obvious symptoms were sore throat (46.15%), followed by fever (34.62%) and cough (30.77%) (**Figure 2A**). Additionally, we evaluated the duration of each symptom in three group, revealing that the reinfection symptoms improved within one week in majority of cases (**Figure 2B**).

Next, an analysis was conducted on reinfected individuals who were severe/critical in primary infection or older than 65. There were 32 WT and 14 Delta cases included. The most common symptoms among this subset of patients included fever, cough, expectoration, and sore throat (**Figure 2C**). Similar to the overall study population, most of reinfection symptoms of these individuals were alleviated within 7 days. However, there was a greater proportion whose clinical symptoms lasted more than 7 days in the WT group than that in Delta group (**Figure 2D**).

### 3.4 SARS-CoV-2 specific humoral and T cells response of participants with reinfection and non-reinfection

**Figure 3:**
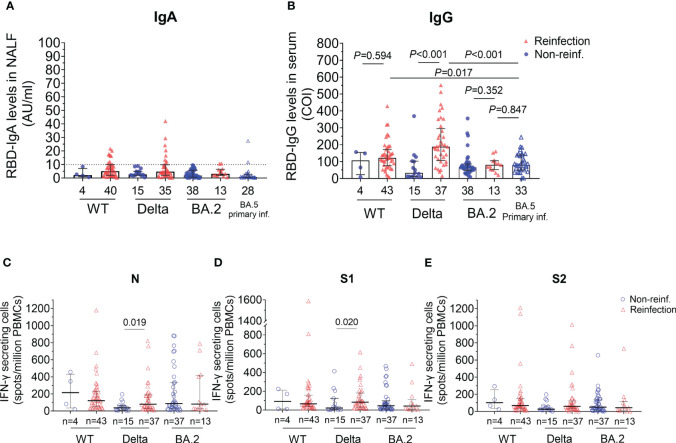
SARS–CoV–2 specific humoral and T cells response of participants with reinfection and non–reinfection. **(A)** Anti–RBD IgA antibody values in nasal lavage fluid. The RBD–IgA was detected by the semi–quantitative method which defines the result greater than 10 AU/ml as positive, bounded by the horizontal dotted line. **(B)** Anti–RBD IgG antibody values in serum. COI (cut off index) represents the ratio of the detected optical intensity value to the threshold value. If COI is less than 1, the result is negative. **(C–E)** Different T cellular response stimulated by three peptide pools. Numbers of IFN–γ–secreting cells per million PBMCs were shown. Statistical significance was determined using Mann–Whitney U–test. WT, wild type. RBD, receptor binding domain. NALF, nasal lavage fluid. PBMCs, peripheral blood mononuclear cells.

Data about the immunity response after reinfections are scarce, especially in China. In this study, we detected anti-RBD IgA/IgG and specific IFN-γ-secreting T cells. Among the reinfected individuals, 20% (8/40) of ancestral virus, 25.7% (9/35) of Delta and 15.4% (2/13) of BA.2 group remained positive for nasal anti-RBD IgA (**Figure 3A**). Meanwhile, we also detected the nasal RBD-IgA of people who infected Omicron BA.5 for the first time, and found that the RBD-IgA (0.7985AU/ml) levels were not significantly higher than that in the Omicron BA.2 reinfection population.

Serum anti-RBD IgG levels were also detected (**Figure 3B**). The results showed that the anti-RBD IgG levels in reinfections were higher than those in non-reinfections in the WT strain group, however, there was no statistically significant difference between the two groups (median 121.26 vs. 106.80, *P*=0.594). Furthermore, in the Delta group, serum anti-RBD IgG levels were significantly higher in the reinfected group than those in the non-reinfected group (median 188.15 Vs. 33.24, *P*<0.001). However, we found serum IgG responses in prior BA.2 infection cases appeared unaffected after BA.5 reinfection (80.21 vs. 66.57, *P*=0.352). Compared with people who were primarily infected with BA.5, the RBD-IgG levels of BA.5 reinfections in WT and Delta group increased significantly but not in BA.2 group (WT, *P*=0.017; Delta, *P*<0.001; BA.2, *P*=0.847, **Figure 3B**).

To analyze T cells response after reinfected with Omicron, we detected the IFN-γ secreting cells after stimulation by peptide pools of nucleocapsid (N), Spike 1 subunit (S1) or 2 (S2). Similar to humoral response, the number of viral-specific IFN-γ secreting T cells of the reinfected individuals was higher than that of non-reinfections in Delta group after stimulation by peptide pools of N and S1 proteins (N, *P*=0.019 and S1, *P*=0.020, **Figures 3C, D**). Of note, viral-specific cellular immune protection persisted after 6-12 months recovered from BA.2 primary infection though without reinfection. However, there were no difference in specific T cell response between reinfections and non-reinfections in BA.2 recovered group, indicating that BA.5 reinfection may not cause strong T cell response in patients primarily infected with BA.2.

## 4 Discussion

This study retrospectively analyzed the severity, outcomes and immune response of reinfection in local cases primary infected with ancestral virus, Delta or Omicron BA.2 in Guangzhou during the BA.5 pandemic from December 2022 to January 2023.

SARS-CoV-2 infection can develop cross-immunity against different variants for a certain time. However, after the emergence of Omicron, which was able to escape from immune surveillance because of multiple spike mutations, increasing numbers of reinfections have been observed (Pulliam et al., 2022). In this study, we found the BA.5 reinfection rates of prior ancestral virus and Delta were significantly higher than that of prior BA.2 infection, indicating that the effectiveness of pre-Omicron primary infection against Omicron reinfection is relatively low. This is consistent with studies from other countries. Studies from Qatar reported that the effectiveness of pre-Omicron primary infection against BA.4 or BA.5 reinfection was only 27.7%, while that of post-Omicron infection against reinfection was 78.0% (Altarawneh et al., 2022), and the effectiveness against reinfection declined with time since primary infection (Chemaitelly et al., 2022). Singapore’s national cohort study also found first infections with BA.2 could provide greater protection against BA.4 or BA.5 or XBB than pre-Omicron variants (Tan et al., 2023), indicating that the protection against SARS-CoV-2 reinfection with the same strain is significantly higher.

In addition, we sought to determine the factors that influence reinfection. Interestingly, we observed a higher incidence of reinfection in women compared to men, particularly in the BA.2 primary infection group, with significant differences. It may be a result of higher exposure among females. A review included 23231 reinfected patients found that females were predominant among reinfections (M/F=0.8) (Flacco et al., 2022; Nguyen et al., 2023). However, not all studies have been able to observe this phenomenon, which may depend on the background of the population observed.

Study from Spain supported that there were higher reinfection rates and lower disease severity during BA.5 than previously variants (Ciuffreda et al., 2023). In this study, only one patient was categorized as medium case, four were asymptomatic and the rest were all mild cases during BA.5 reinfection according to the 10^th^ Diagnosis and Treatment Protocol for COVID-19, none progressed to critical or fatal case. This is consistent with a review reported that only 0.58% manifested as severe illness, and 0.04% manifested as critical illness among those reinfection cases (Chemaitelly et al., 2022). The effectiveness of primary infection against severe, critical or fatal COVID-19 reinfection remains very strong, which reduce the risk of severe illness during reinfection. In addition, we found that fever, cough and sore throat were the most common symptom of BA.5 reinfection regardless of the variant of the first infection. Similar results were found in the severe/critical cases in primary infection or cases older than 65. This can be attributed to the reduced virulence of the Omicron BA.5 (Guo et al., 2023), partly due to less infectivity to the lungs (Halfmann et al., 2022). Because of the early dynamic zero policy, SARS-CoV-2 reinfection was less reported in China. As a result, data on the immunity response after reinfections conferred by earlier SARS-CoV-2 primary infections are scarce. In this study, we found that after the BA.5 reinfection, cases who previously infected with ancestral SARS-CoV-2 or Delta had higher levels of nasal specific IgA, serum specific IgG and T cell immune responses than cases primary infected with BA.2.

It is reported that cases who had higher level of mucosal specific IgA get significantly lower risk of Omicron infection (Havervall et al., 2022) and nasal IgA but not IgG correlates with nasal neutralization after COVID-19 (Wright et al., 2022). Thus, nasal specific IgA can be used as an indicator of the risk of reinfection. In this study, we found all non-reinfected individuals showed negative test of nasal specific IgA (**Figure 3A**) which may be related to the interval since initial infection were over two years for WT group, over 18 months for Delta group and over 6 months for BA.2 group. Felicity Liew et al. had also reported that SARS-CoV-2-specific nasal IgA disappear 9 months after hospitalization (Liew et al., 2023). After reinfection with BA.5, the nasal IgA in reinfected individuals of WT and Delta initial infection group were higher than that in non-reinfected individuals. However, the levels of nasal IgA in previously infected BA.2 cases were similar in both reinfected and non-reinfected individuals.

A similar trend has been observed in serum anti-RBD IgG. Compared with people who were primarily infected with BA.5, the RBD-IgG levels of BA.5 reinfections in WT and Delta group increased significantly but not in BA.2 group. This is consistent with several previous reports of enhanced magnitude of antibody response following exposure to multiple variants (Laurie et al., 2022; Branche et al., 2023). It suggests that exposure to two distant variants has protective potential against emerging variants with some degree of similarity to currently and previously circulating VoCs (Rossler et al., 2022).

We demonstrate that neutralizing antibody responses are strongest against variants sharing certain spike mutations with the immunizing exposure, and exposure to multiple spike variants increases breadth of variant cross-neutralization. One reason may be that BA.5 infections induce lower levels of antibody production (Guo et al., 2023). On the other hand, nasal IgA responses to Omicron are short-lived. The nasal IgA binding to Omicron increased 2-4 weeks post-infection but remain positive only between 3 to 5 months post-infection (Liew et al., 2023). As our tests were performed 3 months after reinfection, during which time the antibody levels may have waned. The short-lived or low humoral response may explain the higher reinfection rates in the Omicron period than in the pre-Omicron period. This could be due to lower immunogenicity and higher immune evasion of BA.5 (Wang et al., 2022). Further research is required to explore.

T cell response is also critical for protection against SARS-CoV-2 infection, especially when antibodies titers wane and variants emerge (Guo et al., 2022; Kedzierska and Thomas, 2022). We found T cell immune response were stronger in reinfected cases than that in non-reinfected ones who primary infected with Delta. Strikingly, after BA.5 reinfection, the specific T cell response and antibody levels were similar in reinfected and non-reinfected groups who were initially infected with BA-2. These results suggest that reinfection with different variants may cause a stronger specific immune response from the host, while reinfection with the same variants of the primary infection cannot strengthen the established immune response of the host. However, we do not know how well these two groups will be able to fight the next possible reinfection, and further follow-up and surveillance may explain this phenomenon.

However, there are several limitations to considerate in this study. Firstly, limited cases. All the individuals were from COVID-19 cases admitted to our hospital in Guangzhou, and only half of them were successfully contacted. There may be some bias in the results, for example older people may have trouble answering the phone. Secondly, the clinical follow-up was carried out three months after reinfection, the symptoms of reinfection depend on patients’ recall and may result in recall bias due to unclear memory or varying seriousness of recall. On the other hand, the test results reflected the state three months after reinfection rather than the initial stage of recovery from reinfection. Thirdly, the RBD antigen used to detect specific IgG and IgA is from the ancestral strain, so the results of BA.2 primary infection were lower. But IgG against Delta and Omicron RBD were correlated with IgG levels against the ancestral strain (Zhang et al., 2023). And the peptide pool used for T cell stimulation in this study is encoded the ancestral strain epitopes only. This may affect our observation of specific T cell responses induced by S protein after Omicron infection as the cross-reactivity between ancestral and omicron Spike immunodominant T-cell epitopes is low. Nevertheless, we can still look at the specific T cell response after stimulation with peptides of nucleocapsid(N) protein which was relatively conserved. And the stimulation of peptides from ancestral SARS-CoV-2 can partly reflect the specific T cell response after mutant strains infection (Bormann et al., 2023). Furthermore, we did not measure immune response of these cases before reinfection, so the immune response of patients after reinfection we showed provided limited information. However, we were sure that the serum level of RBD-IgG in Delta group was low before reinfection, and increased significantly after reinfection (data not show). Lastly, neutralizing antibodies against different Omicron stains were not detected in this study, though RBD binding antibody had a high correlation with neutralizing capacity (Feng et al., 2021).

Populations appear to be generally vulnerable to reinfection by emerging SARS-CoV-2 variants or sub lineages with greater immune-escape capabilities. At present, reinfection by XBB variants has been prevalent in China. Data from Singapore showed that the protection against XBB reinfection from earlier Omicron variant infection was lower and waned faster than that against BA.4 and BA.5, thus result in reinfection rate was higher during the XBB-driven wave than that during the BA.5 wave (Tan et al., 2023). On June 23, 2023, the U.S. CDC reported a study of SARS-CoV-2 reinfections for the first time in a weekly report. As the Omicron BQ.1/BQ.1.1 became dominant, the percentage of reinfections and that of hospitalizations or deaths increased substantially (Ma et al., 2023). It seems difficult to avoid reinfection and we should maintain attention, achieve early detection and early treatment.

In summary, we found that BA.5 reinfection is common but severe outcomes are relatively rare. It is suggested to understand the risk of reinfection scientifically, receive early antiviral treatment when eligible during reinfection and keep ongoing attention to emerging SARS-CoV-2 variants. Reinfection with different SARS-CoV-2 variants from primary infection may cause a stronger immune protection and this should be considered for the development of vaccine.

## Data availability statement

The datasets for this article are not publicly available due to concerns regarding patient anonymity. The raw data supporting the conclusions of this article will be made available by the authors. Requests to access the datasets should be directed to the corresponding author.

## Ethics statement

The studies involving humans were approved by The ethical committee of Guangzhou Eighth People’s Hospital. The studies were conducted in accordance with the local legislation and institutional requirements. Written informed consent for participation in this study was provided by the participants’ legal guardians/next of kin.

## Author contributions

XD: Writing – original draft, Project administration, Funding acquisition, Resources, Supervision, Writing – review & editing. FL: Writing – original draft, Writing – review & editing, Project administration, Supervision. XT: Supervision, Writing – review & editing, Resources, Writing – original draft. LL: Project administration, Formal analysis, Investigation, Methodology, Writing – original draft. ZX: Data curation, Formal analysis, Writing – original draft, Methodology. YL: Data curation, Methodology, Writing – original draft. ML: Formal analysis, Investigation, Project administration, Writing – original draft. LZ: Methodology, Writing – original draft. CF: Methodology, Writing – original draft. SJ: Investigation, Methodology, Writing – original draft. GT: Methodology, Writing – original draft. HH: Project administration, Writing – original draft. RH: Methodology, Writing – original draft. YX: Project administration, Writing – original draft. JS: Data curation, Writing – original draft. QF: Methodology, Writing – original draft. YLi: Resources, Writing – review & editing. QG: Methodology, Writing – original draft. YLa: Resources, Writing – original draft. FH: Resources, Writing – review & editing. NY: Resources, Writing – review & editing.
